# The impact of health information exposure frequency on youth sleep quality under algorithmic recommendation: a serial mediation role of information anxiety and self-efficacy

**DOI:** 10.3389/fpubh.2026.1818140

**Published:** 2026-05-14

**Authors:** Siqi Chen, Jing Cao, Lei Ye, Dong Wei

**Affiliations:** 1 University of Shanghai for Science and Technology, Shanghai, China; 2 Shanghai Publishing and Printing College, Shanghai, China

**Keywords:** algorithmic recommendation, health information, Information anxiety, self-efficacy, serial mediation, sleep quality

## Abstract

**Introduction:**

As algorithmic recommendation technology increasingly dominates the circulation of health information, young people's exposure frequency to relevant content has significantly increased. However, the psychological mechanisms through which this exposure affects sleep quality remain insufficiently understood.

**Methods:**

Based on Social Cognitive Theory and Cognitive Arousal Theory, this study constructs a serial mediation model to investigate how the frequency of health information exposure under algorithmic recommendation influences youth sleep quality through information anxiety and self-efficacy. A questionnaire survey was conducted among individuals aged 18 to 35.

**Results:**

The results show that: (1) the frequency of health information exposure not only directly and negatively predicts sleep quality, but also exerts multiple indirect effects; (2) information anxiety and self-efficacy each serve as independent mediators between information exposure and sleep quality; and (3) information anxiety and self-efficacy form an “emotional-cognitive” serial mediation pathway, whereby greater exposure intensifies information anxiety, undermines self-efficacy, and ultimately impairs sleep quality.

**Discussion:**

By revealing the psychological processes through which the algorithmic information environment affects youth sleep, this study offers theoretical support and practical implications for enhancing digital health literacy, improving platform algorithm governance, and strengthening public health interventions.

## 1 Introduction

Digital technologies have profoundly reshaped the dissemination and access patterns of health information, with algorithmic recommendation systems in social media and news apps becoming the primary channels for young people to obtain health-related knowledge (1). Against this backdrop, algorithms significantly improve users' exposure frequency to health information through precise push notifications, particularly content related to sleep improvement, disease prevention, and nutritional advice (2). However, this high-frequency, personalized, and often contradictory information flow has introduced new public health challenges: On one hand, information overload and cognitive burdens may trigger significant information anxiety (3); on the other hand, sustained exposure to such content may positively or negatively correlate with individuals' confidence in their health management abilities, known as self-efficacy (4). Sleep, a critical indicator for measuring mental wellbeing and quality of life, may systematically be influenced by these emotional and cognitive mechanisms (5).

Although existing studies have confirmed associations between the traditional media usage duration and sleep disorders (6), most studies have been limited to physical factors such as screen time or blue light exposure, failing to elucidate how algorithmic recommendation systems, a novel information context, impact sleep quality through psychological mediation pathways (7). In particular, in the algorithm-driven era “information seeking people”, both the frequency and the mode of health information exposure have undergone fundamental transformations. These shifts call for a systematic examination from an integrated “emotional-cognitive” perspective (8). Consequently, the underlying mechanisms urgently require systematic analysis from an integrated “emotional-cognitive” perspective (8). Therefore, clarifying how the frequency of exposure to health information via algorithmic recommendations is linked to information anxiety, self-efficacy, and sleep quality among young people holds significant theoretical and practical value for unraveling the mechanisms of health behavior formation in the digital age and developing precise sleep health intervention strategies.

## 2 Literature review

A review of existing domestic and international studies shows that most existing studies concentrate on the macro-level relationship between media use and sleep patterns. In contrast, empirical research examining how algorithmic recommendation environments affect youth sleep quality remains limited. In particular, the psychological mechanisms through which such effects occur have not yet been sufficiently explored.

### 2.1 From “People Seeking Information” to “Information Seeking People”: How Algorithms Reshape Health Information Exposure

Algorithmic recommendation systems have reshaped the way health information is distributed. By drawing on users' browsing histories, social interactions, and preference profiles, these systems deliver highly personalized content (9). Studies suggest that this “information seeking people” model has increased young users” exposure to health-related topics and deepened their engagement, particularly in areas such as sleep optimization (1, 10).

However, highly homogenized information streams are prone to creating “Filter Bubble” (9) or “echo chambers” (8), which narrow users” informational horizons and may contribute to cognitive bias (11). Although algorithmic recommendation has been shown to enhance user engagement and improve the efficiency of information access (2), its potential negative effects on psychological states (e.g., anxiety levels) and subsequent health behaviors (e.g., sleep patterns) remain insufficiently examined. Existing research has largely focused on the influence of algorithms on political attitudes (12) or consumer behavior (13). In contrast, their impact within health communication has received far less attention. In particular, the psychological pathways through which algorithmic environments affect basic physiological processes, including sleep, remain largely unexplored.

### 2.2 Formation mechanism of information anxiety and its negative impact on sleep

Exposure to excessive and conflicting health information represents a key psychological mechanism through which information anxiety is triggered and sleep quality is undermined. Information anxiety describes the tension, worry, and sense of helplessness people experience when they are confronted with large volumes of complex or conflicting content that exceeds their ability to process and make sound decisions (3, 14). Within the health information context, core contributors include information overload (15), doubts about source credibility (16), and heightened decision-making pressure (14). According to Cognitive Arousal Theory (17), heightened cognitive activation and emotional stress before bedtime represent key factors disrupting sleep onset and maintenance. Empirical evidence further suggests that health-related content, particularly information involving personal risk, is especially likely to provoke anxiety, which in turn contributes to longer sleep latency, reduced sleep efficiency, and more frequent nocturnal awakenings (18, 19). Notably, during the COVID-19 pandemic, public exposure to massive volumes of conflicting health information created an “infodemic” (20), which significantly exacerbated psychological distress and sleep disturbances (21). Taken together, frequent exposure to algorithm-recommended health information may heighten information anxiety and function as a significant stressor that compromises sleep quality among young people.

### 2.3 Mediating role of self-efficacy: bridging information reception and health behaviors

In the process of information influencing behaviors, individual self-efficacy plays a crucial mediating role. As a core construct of Social Cognitive Theory (4), self-efficacy refers to individuals' confidence in their capability to organize and carry out actions required to achieve specific goals, such as improving sleep quality. Research shows that moderate and high-quality health information exposure can increase knowledge reserves and enhance perceived control, thereby strengthening self-efficacy (22, 23). When information exposure becomes excessive (24), however, or when content generates anxiety and confusion (25), individuals may experience frustration from perceived discrepancies between goals and capabilities. This process ultimately undermines self-efficacy (26). Self-efficacy has been widely recognized as a key cognitive predictor of various health behaviors, including regular sleep schedules and avoidance of pre-sleep stimulation. Moreover, it demonstrates a direct and positive association with sleep quality (27, 28). For instance, a study on insomnia patients found that improving sleep-related self-efficacy through cognitive behavioral therapy led to significant gains in subjective sleep quality (29). Therefore, self-efficacy self-efficacy likely serves as a crucial mediator between health information exposure and sleep quality.

### 2.4 Integrating the cognitive–emotional dual pathways: theoretical foundations for a serial mediation model

Most prior research has tended to approach media effects from a single angle, focusing either on an emotional route (such as anxiety) or a cognitive route (such as self-efficacy), while paying relatively little attention to how these two processes may interact. The Stress–Cognition–Sleep Model (30) proposes that emotional stressors–for instance, information-related anxiety–can gradually weaken relatively stable cognitive resources like self-efficacy, and together these factors shape sleep outcomes. Some preliminary findings from studies on social media use lend support to this perspective. For example, social anxiety has been found to influence psychological wellbeing indirectly by lowering individuals' sense of self-efficacy (31), suggesting a possible sequential process in which information anxiety first affects self-efficacy. Moreover, information-processing theories argue that anxiety occupies limited cognitive resources and interferes with effective problem solving and decision making, which may in turn diminish confidence in carrying out health-related behaviors (32, 33). That said, it remains unclear whether this kind of sequential mediation mechanism also applies in the context of algorithmically recommended health information, and this question still requires empirical investigation.

Taken together, the current body of research points to several important gaps. To begin with, although algorithmic recommendation technologies are known to significantly shape what information people are exposed to (1, 2), their potential impact on young people's sleep quality–particularly through underlying psychological processes–has not been systematically explored. In addition, previous studies have shown that information anxiety (18, 19) and self-efficacy (27, 28) can each serve as mediators linking media use to health-related outcomes. However, an important issue remains insufficiently addressed: might information anxiety and self-efficacy operate sequentially, forming a chain mediation pathway between exposure to algorithmically recommended health information and youth sleep quality? So far, direct empirical evidence for such a mechanism is still limited. To fill these theoretical gaps, this study integrates the three strands of literature and argues for the need to examine, within a unified analytical framework, the frequency of information exposure under algorithmic recommendation, information anxiety, and self-efficacy. Information anxiety is positioned as the first mediator and self-efficacy as the second for the following reasons. Temporally, exposure to information first triggers immediate emotional responses (i.e., anxiety). This emotional state then erodes more stable cognitive resources (i.e., self-efficacy), ultimately affecting behavioral outcomes (i.e., sleep quality). This sequence– “stimulus → emotion → cognition → behavior”–is supported by both cognitive arousal theory and social cognitive theory, each emphasizing different aspects of the process. The former explains how information exposure induces anxiety through an emotional pathway, while the latter clarifies how cognitive resources (self-efficacy) bridge psychological states and health-related behaviors. Accordingly, this study integrates these two theoretical frameworks to construct a chain mediation model involving information anxiety and self-efficacy, with the aim of providing a more comprehensive explanation of the underlying mechanisms.

## 3 Research design

Based on Social Cognitive Theory (4) and Cognitive Arousal Theory (17), this study explores the ways in which the frequency of exposure to health information within algorithmic recommendation environments may affect young people's sleep quality through the roles of information anxiety and self-efficacy. Social Cognitive Theory highlights the triadic reciprocal interaction among environmental factors (algorithm-driven information streams), personal cognition (self-efficacy), and behavior (sleep behavior). It thus accounts for the cognitive pathway: anxiety consumes cognitive resources, which in turn undermines individuals' self-efficacy in managing their health and ultimately affects sleep quality. Cognitive Arousal Theory, by contrast, posits that anxiety arising during information processing can lead to heightened cognitive arousal. In this study, it explains the emotional pathway: frequent exposure to algorithmically recommended health information may induce anxiety, which is associated with increased cognitive activation. Together, these two frameworks provide coherent theoretical support for the proposed “emotion → cognition” sequential mechanism. By integrating them, this study suggests that algorithmic recommendation is negatively associated with sleep quality by triggering information anxiety (emotional pathway) and weakening self-efficacy (cognitive pathway).

### 3.1 Research hypotheses

Before moving forward, it is important to clarify several key concepts. The primary dependent variable in this study is youth sleep quality, which refers to individuals' subjective assessments of their sleep efficiency, sleep duration, and overall sense of restfulness or recovery (34). The independent variable, health information exposure frequency under algorithmic recommendation, denotes how often and how long young people are exposed to health-related content through algorithm-driven platforms such as TikTok (Douyin) and Xiaohongshu (10). The two mediating variables examined in this study are information anxiety (3) and self-efficacy (4).

To better understand how exposure to algorithmically recommended health information may influence youth sleep quality, this study develops a serial mediation model that includes both information anxiety and self-efficacy. Based on this framework, research hypotheses are formulated at four levels: the direct relationship, the emotional pathway, the cognitive pathway, and the integrated (serial) pathway.

#### 3.1.1 Direct effect of health information exposure on sleep quality

According to Cognitive Arousal Theory (17), heightened cognitive activation before sleep interferes with sleep onset and maintenance. By continuously and frequently pushing health-related information, algorithmic recommendation systems may place young people in a state of cognitive overload, particularly during nighttime use, thereby increasing psychological arousal and directly impairing sleep quality (6). Accordingly, this study proposes:

H1: Health information exposure frequency under algorithmic recommendation is significantly negatively correlated with young people's sleep quality.

#### 3.1.2 The mediating role of information anxiety: the emotional pathway

Information anxiety is defined as the tension and sense of helplessness individuals experience when confronted with excessive, complex, or contradictory information (3). According to Cognitive Arousal Theory, anxiety intensifies cognitive activation and disrupts physiological sleep processes (17). Empirical studies have shown that information overload and inconsistency in health information significantly increase users' anxiety levels (21), and that anxiety is closely associated with poorer sleep quality (18). Therefore, this study proposes:

H2: Information anxiety mediates the relationship between health information exposure frequency and youth sleep quality.
H2a: Health information exposure frequency positively predicts information anxiety.
H2b: Information anxiety negatively predicts youth sleep quality.

#### 3.1.3 The mediating role of self-efficacy: the cognitive pathway

Self-efficacy, a central concept in Social Cognitive Theory, refers to an individual's belief in their capacity to carry out specific behaviors successfully (4). Previous research suggests that an appropriate level of exposure to health information may strengthen this sense of confidence (22). However, when individuals are confronted with excessive or inconsistent health information, their confidence in managing their own health can be weakened (24). In addition, self-efficacy has been shown to be an important predictor of sleep quality (27). On this basis, the present study proposes:

H3: Self-efficacy plays a mediating role in the association between the frequency of health information exposure and youth sleep quality.
H3a: A higher frequency of exposure to health information is expected to be associated with lower levels of self-efficacy.
H3b: Higher levels of self-efficacy are expected to be associated with better sleep quality among youth.

#### 3.1.4 The serial mediation of information anxiety and self-efficacy: an integrated emotional–cognitive pathway

Attention Control Theory (32) posits that anxiety draws on limited cognitive resources, weakening executive control and problem-solving capacity, and consequently diminishing individuals' sense of self-efficacy. In the context of social media use, Peter Muris (31) identified a sequential pathway in which anxiety negatively influences self-efficacy, which in turn affects mental health outcomes. In the context of this study, frequent exposure to health information may first increase information anxiety, which then depletes cognitive resources and reduces self-efficacy, ultimately affecting sleep. Thus, this study proposes:

H4: The frequency with which young people are exposed to health information affects their sleep quality through a sequential mediation involving both information anxiety and self-efficacy.

Specifically, frequent exposure to health information is negatively associated with sleep quality by increasing information anxiety, which in turn reduces self-efficacy.

### 3.2 Model building

Drawing on the aforementioned theories and hypotheses, this study proposes a serial mediation model to clarify the mechanisms linking the core variables. The model is specified as follows: the effect of the independent variable “Health Information Exposure Frequency Under Algorithmic Recommendation” on the dependent variable “Youth Sleep Quality” consists of four pathways:

Direct pathway (H1): Health information exposure frequency under algorithmic recommendation is directly negatively correlated with youth sleep quality.

Pathway mediated by information anxiety (H2): Health information exposure frequency under algorithmic recommendation induces information anxiety (H2a), which in turn is negatively correlated with youth sleep quality (H2b).

Pathway mediated by self-efficacy (H3): Health information exposure frequency under algorithmic recommendation diminishes self-efficacy (H3a), which in turn is negatively correlated with youth sleep quality (H3b).

Serial mediation pathway of information anxiety and self-efficacy (H4): Health information exposure frequency under algorithmic recommendation first triggers information anxiety, which subsequently impaires self-efficacy, and is ultimately negatively correlated with youth sleep quality.

**Figure 1:**
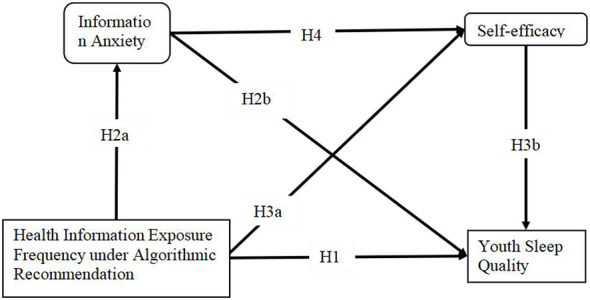
Schematic diagram of the impact of health information exposure frequency under algorithmic recommendation on youth sleep quality.

This model systematically incorporates both affective and cognitive pathways, shedding light on the intricate mechanisms through which the algorithm-recommended information environment affects youth sleep quality. The model structure is illustrated in Figure 1.

### 3.3 Scale design and sample selection

This study adopted a questionnaire survey method for data collection. For the questionnaire, the five-point Likert scale (1 = “Strongly Disagree”, 5 = “Strongly Agree”) was adopted to measure health information exposure frequency, information anxiety, and self-efficacy. Sleep quality was assessed using selected items from the Pittsburgh Sleep Quality Index (PSQI).

Health information exposure frequency: This construct was measured using a scale developed by Nagler and Hornik (10), consisting of three items, including “I encounter health-related information on platforms such as TikTok and Little Red Book every day” and “A large proportion of the health information I obtain is algorithmically recommended.”

Information anxiety: This variable was assessed using a composite scale adapted by Jung and Jung (21) from the Information Overload Scale (15) and the Health Anxiety Scale, comprising three items such as “I feel overwhelmed by the vast amount of health information” and “I worry about missing important health reminders.”

Self-efficacy: This construct was assessed using the Health Self-Efficacy Scale, developed based on the work of Schwarzer and Warner (26), which includes three items such as “I am confident in developing and following a healthy sleep plan for myself” and “Even when confronted with conflicting sleep advice, I am able to judge which option is more suitable for me.”

Sleep quality: This variable was measured using the well-validated Pittsburgh Sleep Quality Index (PSQI) (34), which covers six core dimensions: subjective sleep quality, sleep latency, sleep duration, habitual sleep efficiency, sleep disturbances, and daytime dysfunction. A higher total PSQI score reflects poorer sleep quality among youth.

### 3.4 Data collection and processing

Questionnaires were distributed via online platforms including Credamo and SoJump, targeting young adults aged 18 to 35. A total of 518 responses were collected. After excluding invalid responses (e.g., those with excessively short completion time or patterned answering), 500 valid questionnaires were retained, yielding an effective response rate of 96.5%. All valid data were odellin using SPSS and AMOS, encompassing reliability and validity tests, common method bias assessment (35), correlation analysis, regression analysis, and structural equation odelling (SEM). The significance of mediating effects was examined using the Bootstrap method with 5,000 resamples (36).

## 4 Data analysis

### 4.1 General profile of youth sleep quality scores

As shown in Table 1, demographic variables have significant effects on sleep quality. In terms of age, the 25–29 group reported the best sleep quality (*M* = 2.72), significantly better than the 18–24 group (*M* = 3.63), a difference that may be attributed to greater lifestyle regularity among those in their late twenties. Gender differences were particularly pronounced: females reported significantly better sleep quality (*M* = 2.30) than males (*M* = 3.90), reflecting inherent gender differences in sleep physiology and behavioral patterns. The relationship between education level and sleep quality was non-linear: participants with a bachelor's degree reported the poorest sleep quality (*M* = 3.85), potentially due to peak academic stress. Social media usage duration was negatively associated with sleep quality; those using social media 3–4 h daily reported the worst sleep quality (*M* = 4.56), which confirms the adverse effect of excessive screen exposure on sleep. Regarding occupational status, the “Other” category (which may include freelancers) exhibited the most severe sleep problems (*M* = 4.92), whereas students reported relatively better sleep quality (*M* = 2.88). Participants with a diagnosed sleep disorder demonstrated significantly poorer sleep quality. These findings reveal the multidimensional factors influencing sleep quality and provide empirical evidence for sleep interventions. These six variables were subsequently included as control variables in the analysis.

**Table 1:** Group differences in sleep quality by demographic variables.

Item	Category	n	Mean	Standard deviation	T/F value
Age	18–24 years	260	3.63	1.54	20.060^***^
	25–29 years	216	2.72	1.58	
	30–35 years	24	3.17	1.47	
Gender	Female	186	3.80	0.49	−4.837^***^
	Male	176	3.51	0.63	
Education level	High school or below	24	2.50	1.81	54.163^***^
	Associate degree	61	2.24	1.64	
	Bachelor's degree	305	3.85	1.38	
	Master's degree or above	110	2.14	1.22	
Social media usage duration	Less than 1 hours	51	2.10	1.47	22.490^***^
	1–2 hours	212	2.88	1.71	
	2–3 hours	112	3.39	1.45	
	3–4 hours	43	4.56	0.21	
	More than 4 hours	82	3.84	1.34	
Occupational status	Student	105	2.88	1.43	8.325^***^
	Employed	342	3.22	1.66	
	Unemployed/Seeking	36	3.37	1.54	
	Other	17	4.92	0.09	
Diagnosed sleep disorder	Yes	35	4.28	1.06	17.922^***^
	No	431	3.05	1.62	
	Uncertain	34	4.25	1.14	

^***^P < 0.001, ^**^P < 0.01, ^*^P < 0.05.

The sleep quality score was the mean value of the total PSQI scores, with a higher score indicating poorer sleep quality.

### 4.2 Reliability and validity tests

#### 4.2.1 Reliability test

This study assessed the reliability of the core latent variables in the proposed model. As reported in Table 2, Cronbach's α coefficients for all dimensions exceeded 0.70, meeting the acceptable reliability threshold in social science research. These results indicate strong internal consistency across measurement items and high data reliability.

**Table 2:** Reliability coefficients for the short-form video scale.

Dimension	Number of items	Cronbach's α
Health information exposure frequency	3	0.93
Information anxiety	3	0.95
Self–efficacy	3	0.96
Sleep quality	6	0.98

#### 4.2.2 Validity test

Exploratory factor analysis was conducted using SPSS to evaluate the validity of the scales. The results are presented in Table 3.

**Table 3:** Validity test of the scales.

KMO measure of sampling adequacy		.962
Bartlett's Test of Sphericity	Approx. Chi–Square	6936.746
	Degrees of Freedom	36
	Sig.	.000

Table 3 Validity Test of the ScalesThe results indicate that the KMO value is 0.962, substantially exceeding the recommended threshold of 0.80, indicating strong partial correlations among variables, high sampling adequacy, and excellent suitability for factor analysis. Moreover, Bartlett's test of sphericity was significant (χ^2^ = 6,936.746, df = 36, *P* < .001), confirming significant correlations among all items and demonstrating high structural validity of the data.

### 4.3 Correlation analysis

Health information exposure frequency exhibited a strong positive correlation with information anxiety (*r* = 0.94) and a strong negative correlation with self-efficacy (*r* = −0.93), suggesting that frequent exposure to algorithm- recommended health information may significantly increase users' anxiety and exert a negative effect on their self-management confidence. Detailed results are shown in Table 4. Notably, information anxiety was strongly positively correlated with sleep quality (*r* = 0.94), while self-efficacy was strongly negatively correlated with sleep quality (*r* = −0.96). These findings further confirm the substantial influence of these two psychological mechanisms on sleep and suggest that algorithm-recommended health information may systematically affect youth sleep quality through both affective and cognitive pathways.

**Table 4:** Descriptive statistics and correlation analysis of core variables.

Variable	Mean	Standard deviation	1	2	3	4
1. Health information exposure frequency	3.39	1.38	1			
2. Information anxiety	3.35	1.49	0.94^***^	1		
3. Self–efficacy	2.65	1.59	−0.93^***^	−0.95^***^	1	
4. Sleep quality	3.21	1.62	0.92^***^	0.94^***^	−0.96^***^	1

^***^ *P* < 0.001, ^**^ *P* < 0.01, ^*^ *P* < 0.05.

1. the frequency of exposure to health information was calculated as the mean of Q7–Q9. 2. Information Anxiety was calculated as the mean of Q10–Q12. 3. Self–efficacy was calculated as the mean of Q13–Q15 after reverse scoring. 4. Sleep Quality was calculated as the mean of Q16–Q21; a higher score indicates poorer sleep quality.

Notes: 1. the frequency of exposure to health information was calculated as the mean of Q7-Q9. 2. Information Anxiety was calculated as the mean of Q10-Q12. 3. Self-efficacy was calculated as the mean of Q13-Q15 after reverse scoring. 4. Sleep Quality was calculated as the mean of Q16-Q21; a higher score indicates poorer sleep quality.

To address the issue of discriminant validity, this study adopted the Fornell-Larcker criterion for verification. The results (Table 5) showed that the square roots of the average variance extracted (AVE) for each construct (Health Information Exposure Frequency: 0.967; Information Anxiety: 0.976; Self-efficacy: 0.979; Sleep Quality: 0.992) were all greater than the correlation coefficients between the respective construct and other constructs, which indicated that the discriminant validity of the four constructs was established in accordance with the Fornell-Larcker criterion.

**Table 5:** Analysis results of the Fornell–Larcker criterion.

Variable	info_exposure	info_anxiety	self_efficacy	sleep_quality
info_exposure	0.967	0.935	−0.927	0.916
info_anxiety	0.935	0.976	−0.954	0.944
self_efficacy	−0.927	−0.954	0.979	−0.955
sleep_quality	0.916	0.944	−0.955	0.992

Criterion for Judgment: The values on the diagonal (square root of AVE) should be greater than the other values (correlation coefficients) in the corresponding row/column.

However, given the small margin of the square roots of the AVE values, this study further adopted the more stringent Heterotrait-Monotrait (HTMT) ratio criterion for an additional test. The results (Table 6) indicated that the HTMT ratios for most construct pairs exceeded the recommended threshold of 0.90, particularly that between information anxiety and self-efficacy (HTMT = 0.999). This reveals that although each construct has a clear theoretical distinctiveness, there is a high degree of empirical overlap among these constructs in the present sample.

**Table 6:** HTMT test analysis results.

Variable	info_exposure	info_anxiety	self_efficacy	sleep_quality
info_exposure	1.000	0.990	−0.979	0.923
info_anxiety	0.990	1.000	−0.999	0.959
self_efficacy	−0.979	−0.999	1.000	−0.973
sleep_quality	0.923	0.959	−0.973	1.000

Criterion for Judgment: The HTMT ratio should be less than 0.85 (or 0.90).

Several factors may account for the aforementioned phenomenon. First, all variables in this study were measured via self-report and data were collected at a single time point, which may have artificially inflated the correlations among variables (35). Second, from a theoretical perspective, information anxiety and self-efficacy represent a highly coupled psychological process in the context of health information. Both the cognitive arousal theory (17) and the attention control theory (32) point out that anxious emotions directly deplete self-regulatory resources, and thus the high correlation between the two constructs is theoretically justified. Third, each scale consists of only three items, and short scales tend to exhibit higher inter-construct correlations when they have high reliability. Despite the aforementioned empirical overlap, the Fornell-Larcker criterion is still satisfied, and the core contribution of this study lies in revealing the process mechanism of serial mediation rather than merely examining the distinctiveness between bivariate constructs.

### 4.4 Mediation effect test

Table 7 shows that, after controlling for the influence of demographic variables (age, gender, education level, duration of social media use, employment status and history of sleep disorders), Model 1 indicates that health information exposure frequency significantly and positively predicted information anxiety (β = 0.938, *t* = 51.319, *P* < 0.001). In Model 2, when both health information exposure frequency and information anxiety were included as predictors, they both significantly and negatively predicted self-efficacy (β_exposure = −0.348, *t* = −8.597, *P* < 0.001; β_anxiety = −0.698, *t* = −17.597, *P* < 0.001), indicating that both information exposure and information anxiety reduce an individual's self-efficacy. In Model 3, health information exposure frequency significantly and positively predicted deterioration in sleep quality (β = 0.981, *t* = 42.629, *P* < 0.001). However, in the full Model 4, after controlling for information anxiety and self-efficacy, the direct effect of health information exposure frequency on sleep quality became non-significant (β = 0.070, *t* = 1.642, *P* > 0.05). In contrast, information anxiety significantly and positively predicted the deterioration in sleep quality (β = 0.321, *t* = 6.443, *P* < 0.001), while self-efficacy significantly and negatively predicted sleep quality deterioration (β = −0.608, *t* = −13.716, *P* < 0.001). This indicates that higher self-efficacy is associated with better sleep quality.

**Table 7:** Test of the mediation effect model.

Variable	info_anxiety	self_efficacy	sleep_quality	sleep_quality
constant	0.384 (1.706)	7.342^**^ (36.917)	0.149 (0.525)	4.327^**^ (11.399)
Q1	0.000 (0.002)	−0.105^**^ (-2.834)	−0.080 (-1.505)	−0.143^**^ (-3.916)
Q2	−0.410^**^ (-8.662)	0.114^*^ (2.553)	−0.549^**^ (-9.225)	−0.175^**^ (-3.954)
Q3	0.118^**^ (3.697)	0.013 (0.457)	−0.046 (-1.145)	−0.126^**^ (-4.505)
Q4	0.083^**^ (4.275)	−0.029 (-1.690)	0.013 (0.523)	−0.067^**^ (-3.905)
Q5	−0.095^*^ (-2.250)	−0.069 (-1.866)	0.104^*^ (1.966)	0.132^**^ (3.610)
Q6	−0.014 (-0.219)	−0.499^**^ (-8.608)	0.274^**^ (3.302)	−0.019 (-0.309)
info_exposure	0.938^**^ (51.319)	−0.348^**^ (-8.597)	0.981^**^ (42.629)	0.070 (1.642)
info_anxiety		−0.698^**^ (-17.597)		0.321^**^ (6.443)
self_efficacy				−0.608^**^ (-13.716)
sample size	500	500	500	500
R 2	0.901	0.933	0.867	0.938
Adjusted R 2	0.9	0.932	0.865	0.936
F value	F (7,492) = 640.271, *P* = 0.000	F (8,491) = 853.539, *P* = 0.000	F (7,492) = 458.076, *P* = 0.000	F (9,490) = 817.327, *P* = 0.000

^*^P < 0.05, ^**^P < 0.01, values in parentheses are *t*-values.

The analysis of Table 8 indicates that the effect of health information exposure frequency on sleep quality is fully mediated through a chain mediation mechanism involving information anxiety and self-efficacy. Health information exposure significantly increases information anxiety (Effect = 0.938, *P* < 0.001) and reduces self-efficacy (Effect = −0.348, *P* < 0.001). Furthermore, information anxiety further decreases self-efficacy (Effect = −0.698, *P* < 0.001) and worsens sleep quality (Effect = 0.321, *P* < 0.001), while self-efficacy significantly improves sleep quality (Effect = −0.608, *P* < 0.001). After controlling for these mediating variables, the direct effect of health information exposure on sleep quality is not significant (Effect = 0.07, *P* = 0.101), while the total effect is highly significant (Effect = 0.981, *P* < 0.001), confirming the presence of a full mediation effect.

**Table 8:** Summary of the effect analysis process.

Effect	Term	Effect	SE	*t*	*P*	LLCI	ULCI
Direct effect	info_exposure⇒sleep_quality	0.07	0.043	1.642	0.101	−0.014	0.154
Indirect effects	info_exposure⇒info_anxiety	0.938	0.018	51.319	0	0.902	0.974
	info_exposure⇒self_efficacy	−0.348	0.041	−8.597	0	−0.428	−0.269
	info_anxiety⇒self_efficacy	−0.698	0.04	−17.597	0	−0.776	−0.62
	info_anxiety⇒sleep_quality	0.321	0.05	6.443	0	0.223	0.418
	self_efficacy⇒sleep_quality	−0.608	0.044	−13.716	0	−0.695	−0.521
Total effect	info_exposure⇒sleep_quality	0.981	0.023	42.629	0	0.936	1.026

LLCI denotes the lower bound of the 95% confidence interval for the estimate, while ULCI denotes the upper bound of the 95% confidence interval for the estimate.

This finding reveals a key psychological mechanism in the era of digital health: excessive exposure to health information does not directly damage sleep, but rather is subtly associated with the decline of youth sleep quality through anxiety and negative correlation coping abilities. Intervention strategies should focus on enhancing health information literacy to reduce information anxiety while strengthening self-efficacy, particularly by building psychological resilience in information-overloaded environments. Self-efficacy, as the strongest predictor of sleep quality, offers the most promising intervention target. This suggests that professionals should integrate cognitive-behavioral interventions with sleep hygiene education to systematically enhance individuals' psychological regulation capabilities within health information environments.

**Figure 2:**
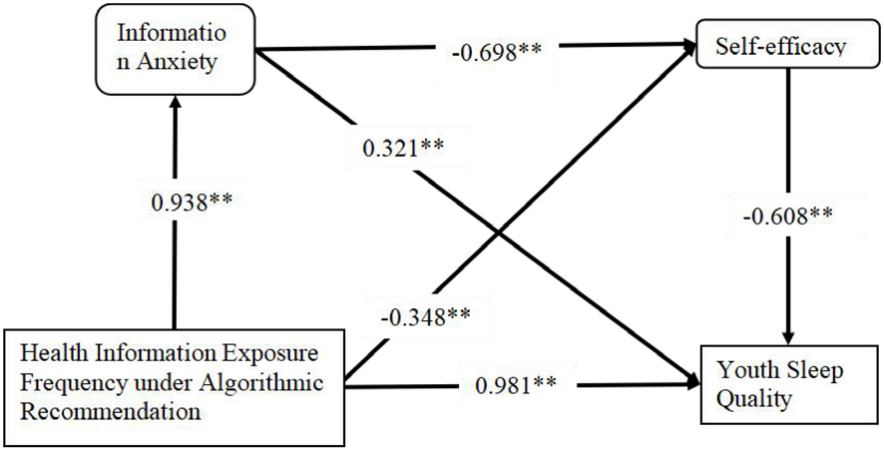
Information anxiety and self-efficacy on the relationship between health information exposure frequency and sleep. *P < 0.05; **P < 0.01.

The analysis of Tables 9, 10 reveals that the impact of health information exposure frequency on sleep quality is fully mediated through a chain mediation mechanism involving information anxiety and self-efficacy. The specific pathways through which health information exposure frequency influences sleep quality are illustrated in Figure 2.

**Table 9:** Summary of the effect analysis process.

Effect	Term	Effect	SE	*t*	*P*	LLCI	ULCI
Direct effect	info_exposure⇒sleep_quality	0.07	0.043	1.642	0.101	−0.014	0.154
Indirect effects	info_exposure⇒info_anxiety	0.938	0.018	51.319	0	0.902	0.974
	info_exposure⇒self_efficacy	−0.348	0.041	−8.597	0	−0.428	−0.269
	info_anxiety⇒self_efficacy	−0.698	0.04	−17.597	0	−0.776	−0.62
	info_anxiety⇒sleep_quality	0.321	0.05	6.443	0	0.223	0.418
	self_efficacy⇒sleep_quality	−0.608	0.044	−13.716	0	−0.695	−0.521
Total effect	info_exposure⇒sleep_quality	0.981	0.023	42.629	0	0.936	1.026

**Table 10:** Indirect Effect Analysis.

Term	Effect	Boot SE	BootLLCI	BootULCI	z	*P*
Info_exposure⇒info_anxiety⇒sleep_quality	0.301	0.05	0.156	0.352	6.054	0
Info_exposure⇒self_efficacy⇒sleep_quality	0.212	0.027	0.129	0.235	7.848	0
Info_exposure⇒info_anxiety⇒self_efficacy⇒sleep_quality	0.398	0.028	0.278	0.389	14.125	0

BootLLCI denotes the lower bound of the 95% bootstrap sampling interval, BootULCI denotes the upper bound of the 95% bootstrap sampling interval, bootstrap type: percentile bootstrap method.

The test of the direct effect reveals that, after controlling for mediating variables, the direct effect of health information exposure on sleep quality is not significant [Effect = 0.07, SE = 0.043, *t* = 1.642, *P* = 0.101, 95% CI (-0.014, 0.154)]. However, the total effect is large and significant [Effect = 0.981, SE = 0.023, *t* = 42.629, *P* < 0.001, 95% CI (0.936, 1.026)], indicating that the mediating variable fully explains this relationship.

Bootstrap analysis of indirect effects further confirms three significant mediating pathways: (1) health information exposure → information anxiety → sleep quality [Effect = 0.301, BootSE = 0.05, 95% CI (0.156, 0.352)]; (2) health information exposure → self-efficacy → sleep quality [Effect = 0.212, BootSE = 0.027, 95% CI (0.129, 0.235)]; and (3) the chain mediation pathway: health information exposure → information anxiety → self-efficacy → sleep quality [Effect = 0.398, BootSE = 0.028, 95% CI (0.278, 0.389)]. The sum of the effect sizes for these three pathways (0.911) approximates the total effect (0.981), confirming the validity of the full mediation model. Specifically, the chain mediation pathway makes the most significant contribution, revealing that health information exposure first increases information anxiety (Effect = 0.938, *P* < 0.001), which then lowers self-efficacy (Effect = −0.698, *P* < 0.001). Self-efficacy, in turn, emerges as the strongest predictor of sleep quality (Effect = −0.608, *P* < 0.001). These findings collectively indicate that the impact of health information exposure on sleep quality is entirely mediated by psychological mechanisms, rather than through a direct effect.

## 5 Results and discussion

This section aims to systematically present and thoroughly examine the core findings of this study. Through rigorous validity and reliability testing of the collected valid sample data, control for common method bias, and structural equation modeling analysis, we validated the chain mediation model demonstrating how the health information exposure frequency under algorithmic recommendations affects youth sleep quality. The following will first summarize the research findings, then place them within a broader theoretical and practical context for a multi-level, multi-dimensional discussion. This will clarify their theoretical contributions and practical implications, while reflecting on the limitations of the study to indicate future directions.

### 5.1 In-depth summary and theoretical interpretation of research findings

This study comprehensively validated the proposed theoretical model through structural equation modeling and Bootstrap mediation testing. Data analysis results supported all research hypotheses, revealing the complex pathway through which health information exposure frequency under algorithmic recommendations affects youth sleep quality via a dual “affective-cognitive” mechanism.

Specifically, Hypothesis H1 is supported, there is a significant negative correlation between health information exposure frequency under algorithmic recommendation and youth sleep quality. This finding corroborates cognitive activation theory (17), indicating that under the “information seeking out users” paradigm, high-frequency, passive health information flow itself can constitute cognitive load. This directly disrupts young people's mental tranquility and is correlated with the decline of sleep quality (6).

Second, Hypotheses H2 and H3 hold true, this confirms the independent mediating effects of information anxiety and self-efficacy. The pathway H2 indicates that the health information exposure exhibits a negative correlation with youth sleep quality by triggering information anxiety. This pathway clearly illustrates the “affective pathway” from information overload to emotional distress, and ultimately to physiological sleep disorders. This perfectly aligns with Harvey's (17) cognitive activation theory, which posits that anxiety triggered by information before bedtime leads to cognitive activation, thereby inhibiting the natural relaxation process required for sleep (18, 19). The H3 pathway indicates that the health information exposure frequency exhibits a negative correlation with youth sleep quality by weakening self-efficacy. This demonstrates the mechanism of the “cognitive pathway.” According to Social Cognitive Theory (4), when health information is overly complex or confusing, it can weaken young people's confidence in their ability to manage their own health (self-efficacy) (24, 25). Individuals with lower self-efficacy are more likely to abandon efforts when facing sleep difficulties, leading to poorer sleep quality (27, 28).

The core contribution of this study is to verify hypothesis H4, that is, information anxiety and self-efficacy constitute a continuous “emotion cognition” chain mediation path. This chain mechanism organically integrates cognitive activation theory (17) and social cognitive theory (4), and provides a more refined and dynamic perspective to explain how the algorithmic recommendation environment systematically affects sleep health of young people. Specifically, high-frequency exposure to algorithm-recommended health information is first positively associated with the level of information anxiety (an emotional response). Then, according to the attention control theory (32), this emotional response occupies substantial cognitive resources and is negatively associated with an individual's self-efficacy, ultimately demonstrating a significant negative correlation with young people's sleep quality. The discovery of the complete mediation effect of this path is more theoretically illuminating. One explanation for this finding is that health information contact itself is basically harmless to sleep quality. Only when such contact triggers adverse psychological states, specifically an increase in information anxiety and a decrease in self-efficacy, does sleep quality deteriorate. This means that interventions targeting psychological mediators (rather than simply reducing information exposure) may be sufficient to protect sleep quality. Another explanation is that the influence of psychological mechanism is so powerful that it completely absorbs the part that might have been shown as a direct effect, which indicates that the emotional and cognitive consequences of information contact constitute the main channel of action. Regardless of which interpretation, the results indicate that the key focus of intervention lies in the psychological processing of health information rather than exposure itself, which further highlights the importance of enhancing young people's psychological resilience and information literacy in an algorithm-driven information environment.

Combined with the above conclusions, this study not only confirms the negative correlation between the health information contact frequency on youth's sleep quality, but also reveals its influence mechanism through the independent mediation of information anxiety, the independent mediation of self-efficacy, and the chain mediation of “information anxiety to self-efficacy”. This integration model deepens our understanding of the relationship between the digital information environment and youth health, and highlights the importance of paying attention to the young people's psychological resilience and information literacy in the era of algorithm dominance.

### 5.2 Practical implications and multidimensional strategies based on research findings

To build on these conclusions, this study offers not only theoretical significance but also concrete, pressing actionable insights for young adults, internet platforms, public health policymakers, and clinical practitioners alike.

#### 5.2.1 Cultivating critical digital health literacy among young people

Young people need to change from passive information consumers to active and critical information managers. Digital health literacy should be improved, that is, the ability to obtain, understand, evaluate and apply health information to make beneficial decisions (37). The education system and social organizations should cooperate to carry out the integration education project of media literacy and health literacy, teach the youth to recognize the existence of the algorithm “information cocoons”, critically evaluate the source credibility of health information, and understand the basic logic of algorithm operation to eliminate its “black box” mystery.

Secondly, young people should consciously carry out “information diet” and “digital detoxification”. This includes setting the time limit for using algorithm recommended applications every day, creating a “screen-free” environment at least 1 h before going to bed, and actively subscribing to and trusting a few authoritative sources rather than relying solely on the passive feeding of the algorithm. The digital health literacy scale developed by van der Vaart & Drossaert can be used as a self-assessment tool to help young people understand their shortcomings (37). In addition, the techniques of mindfulness meditation and cognitive behavioral therapy can also be used to manage information anxiety.

#### 5.2.2 Promoting responsible and transparent algorithm design

Internet platforms ought to embrace their social responsibility for algorithmic systems and put user wellbeing at the heart of their design principles.

Introduction of exposure diversity mechanism: the platform should optimize the recommendation algorithm to avoid excessive and homogeneous push on a single health topic. We can learn from the “exposure diversity” framework proposed by Helberger, Karppinen & D'Acunto, embed the diversity of content sources, views and types into the recommendation system, and take the initiative to break the information cocoons (38).

Enhance user control and transparency: provide more refined user control functions, such as the “push fatigue control” slider, allowing users to adjust the push frequency of specific types of information by themselves; Set the “one key pause recommendation” button; And provide a clearer explanation of “why see this content” to increase the transparency of the algorithm (39).

Give priority to push enabling content: in terms of the distribution weight of health information, the platform should give priority to recommending content with authoritative sources, gentle language, operable suggestions and enhanced user self-efficacy. This is in line with Fogg's behavior model, that is, to guide and promote users' positive behavior change through design (40).

#### 5.2.3 Building a public environment supportive of youth sleep health

Public health authorities and clinicians ought to regard the digital information environment as an emerging determinant of population health.

Optimize public health communication strategies: when using new media for health education, health authorities should refrain from fear appeals and information panic. Communication efforts should prioritize simple, clear, consistent and actionable core messages, with the goal of improving public self-efficacy (41).

Integrate information environment assessment into clinical practice: when treating young people with sleep problems, psychologists and sleep therapists should routinely assess their digital media use and exposure to health-related information. Cognitive Behavioral Therapy for Insomnia (CBT-I) is an evidence-based treatment (42) and can be adapted to target cognitive distortions caused by information anxiety.

Advance cross-sectoral regulation and collaboration: governmental regulators should develop guiding principles to hold platforms accountable for their social responsibility in health communication, and curb the spread of misleading and harmful information. Meanwhile, interdisciplinary research on “algorithms and mental health” should be encouraged to provide an evidence base for targeted governance.

### 5.3 Research limitations and future directions

This study has some limitations, which need to be improved in the future. First, it is difficult to establish a strict causal relationship for cross-sectional data. In the future, the empirical sampling method advocated by Bolger & laurenceau (43) or combined with objective measurement tools [such as activity recorder (44)] can be used for dynamic tracking. Secondly, there are relatively high correlation coefficients among the core constructs of this study. HTMT analysis results show that the discriminant validity of some construct pairs did not meet strict threshold standards. Although the Fornell-Larcker criterion was satisfied and the high correlations are reasonable in theory, future research should consider using more items and more differential measurement tools, or using multiple methods (such as physiological indicators combined with self-report) to reduce common method bias and enhance the discrimination between constructs. Finally, this study focuses on the frequency of information contact. In the future, we should deeply analyze the regulatory role of the characteristics of information content (such as the comparison of threatening information and enabling information), and use the extended parallel process model (45) for comparison. Similarly, individual traits [such as neuroticism (46)] may regulate the mediation path, which can be incorporated into the model to identify high-risk people in the future, so as to achieve more precise intervention.

### 5.4 Conclusion

In an era where algorithms increasingly dominate our information diet, this study reveals how exposure to algorithm-recommended health information is associated with poorer sleep quality among young people through the chain mediation of “information anxiety” and “self-efficacy”. This is not only a communication or psychological issue, but also a comprehensive issue related to public health and social welfare. Our findings emphasize that the algorithm is no longer a neutral technical tool, but an environmental force that can systematically shape users' emotional, cognitive and even physiological states.

In the face of this challenge, the efforts of any single subject are weak. It calls for a concerted action: young individuals need to improve their psychological resilience and critical literacy in the digital age; The scientific and technological platform must go beyond the logic of efficiency first and embed the ethical principles of “no harm” and “promoting wellbeing” into the algorithm design; Policy making and clinical practice need to keep pace with the times, and digital environmental health impact assessment should be included in the scope of routine work. Only through the multiple collaborative governance of individuals, technology and society can we control the wave of technology, turn it into an effective tool to improve public health, rather than the source of health problems, and finally guard the sleep health and overall wellbeing of the young generation in the digital age.

## Data Availability

The raw data supporting the conclusions of this article will be made available by the authors, without undue reservation.
